# Preparation of 2D Materials and Their Application in Oil–Water Separation

**DOI:** 10.3390/biomimetics8010035

**Published:** 2023-01-15

**Authors:** Jie Li, Yushan Li, Yiyi Lu, Yuke Wang, Yunjie Guo, Wentian Shi

**Affiliations:** School of Artificial Intelligence, Beijing Technology and Business University, Beijing 100048, China

**Keywords:** two-dimensional materials, oil–water separation, special wettability

## Abstract

The problems of environmental pollution are increasingly severe. Among them, industrial wastewater is one of the primary sources of pollution, so it is essential to deal with wastewater, especially oil and water mixtures. At present, biomimetic materials with special wettability have been proven to be effective in oil-water separation. Compared with three-dimensional (3D) materials, two-dimensional (2D) materials show unique advantages in the preparation of special wettable materials due to their high specific surface area, high porosity, controlled structure, and rich functional group rich on the surface. In this review, we first introduce oil–water mixtures and the common oil–water separation mechanism. Then, the research progress of 2D materials in oil–water separation is presented, including but not limited to their structure, types, preparation principles, and methods. In addition, it is still impossible to prepare 2D materials with large sizes because they are powder-like, which greatly limits the application in oil–water separation. Therefore, we provide here a review of several ways to transform 2D materials into 3D materials. In the end, the challenges encountered by 2D materials in separating oil–water are also clarified to promote future applications.

## 1. Introduction

With industry development, human pollution of the environment is getting worse [1], and this pollution has triggered a series of environmental problems, such as global warming, freshwater resource crisis, land desertification, etc. [2,3,4]. In industrial pollution, wastewater is a problem that cannot be ignored [5]. Once a large amount of wastewater enters the ocean, lakes, and groundwater, it will cause severe damage to the local ecology and human society, and this destruction is often irreversible [6,7,8]. Therefore, strict treatment must be performed before discharging wastewater [9]. Among them, the separation of oil and water is significant because a small amount of oil can pollute a large amount of water.

For this reason, people have adopted many oil–water separation methods, such as membrane separation and sponge adsorption. After the “self-cleaning effect” of lotus leaves was revealed [10,11,12], biomimetic materials with special wettability inspired by nature provided a new direction for oil–water separation. With the emergence of graphene, 2D materials have sparked widespread interest [13,14]. Due to their special structure and properties, 2D materials have shown application potential in different fields [15,16,17,18,19]. With the deepening of research, people find that the thickness of the atomic level and the large specific surface area of 2D materials are very suitable for separating oil and water. Compared with other dimensions, the large specific surface area of two-dimensional materials can bring greater molecular adsorption capacity to oil–water separation materials, making them ideal materials for gas separation and water treatment. In addition, 2D materials can acquire recognition ability after suitable chemical modification, which can selectively repel oil or water in an oil–water mixture. Many active points on the surface of 2D materials also make it easier to prepare special wettable materials, which have been proven to be ideal materials for separating oil–water mixtures [20,21]. Finally, other oil–-water separation materials generally cannot balance high selectivity and permeability. In contrast, the ultra-fast mass transfer and high permeability of 2D materials can break through the opposition between selectivity and permeability, providing new opportunities for efficient oil–water separation materials.

Although 2D materials show excellent potential, they are still in the initial stage of oil–water separation and lack a comprehensive review. We provide here a review of the fabrications, classification, structures, and mechanisms in the oil–water separation of 2D materials on the basis of the recent progress of the research in the field of oil–water separation. In addition, considering the application requirements, several methods for converting 2D materials into 3D materials are also introduced. Finally, the challenges and opportunities encountered by 2D materials in separating oil–water are also clarified to promote future applications.

## 2. Classification and Separation Mechanisms of Oil–Water Mixtures

There are many methods for oil–water separation. In recent years, materials with special wettability are widely considered the most potential separation materials [22,23]. In addition, 2D materials are mostly used to achieve oil–water separation by constructing surfaces with opposite wettability to oil and water [24]. Therefore, this part briefly introduces the types of oil–water mixtures and the action mechanism of materials with special wettability on oil–water mixtures.

### 2.1. Classification

The oil in oil–water separation usually refers to crude oil, mineral oil, gasoline, and other substances containing many alkanes. The oil–water separation mixtures are divided into oil–water immiscible liquid and emulsion, among which oil-water immiscible liquid is the most common. For simple oil–water immiscible liquid, there is always a clear dividing line between water and oil, regardless of the water-to-oil ratio. Therefore, separating the oil–water immiscible liquid is easier, and even traditional separation methods can achieve good results. In contrast, separating oil–water emulsions without boundary lines is much more difficult. In the oil–water emulsion, the water phase or the oil phase is stably dispersed in another phase in the form of tiny droplets so that the oil–water emulsion can be divided into two types: water-in-oil emulsion and oil-in-water emulsion. In addition, the oil–water emulsion that can remain stable for a long time usually contains a certain amount of surfactant.

### 2.2. Separation Mechanism

**Figure 1:**
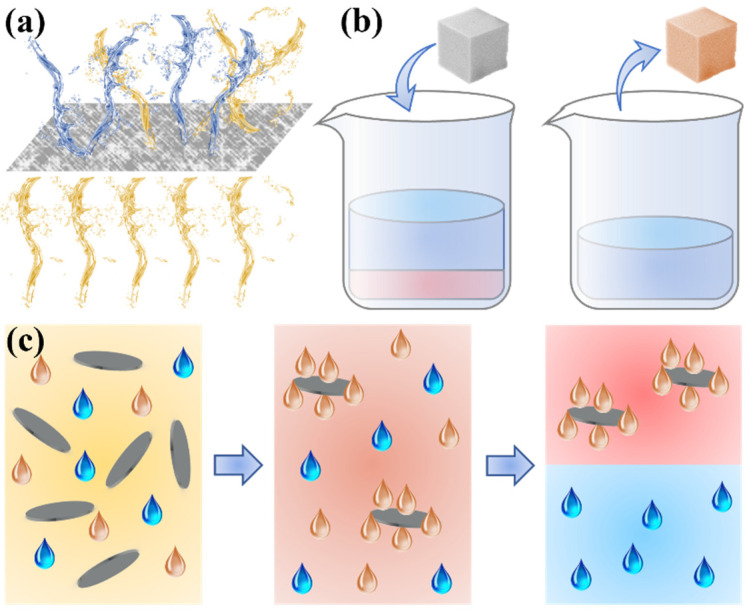
Three action mechanisms of special wettability materials on oil–water mixtures. (**a**) Separation materials. (**b**) Adsorption materials. (**c**) Demulsifiers.

In oil–water separation, special wettable materials can be divided into three types: separation materials, adsorption materials, and demulsifiers, as shown in Figure 1. The surface of the separation materials has opposite wettability to oil and water, and this wettability can selectively intercept one phase of oil or water while the other phase can pass normally. For example, membranes with superhydrophobicity allow the oil phase to pass but not the water phase. The action mechanism of adsorption materials is similar to separation materials, except that a phase through the material’s surface is retained within the material, and this process is generally reversible. That is, under the action of external force, the water or oil absorbed in the material can be discharged. Different from the above two materials, demulsifiers generally exist in the form of powder and can only achieve the demulsification of oil–water emulsions. After dispersing the demulsifiers in oil–water emulsions, they can adsorb the oil phase or the water phase on the material’s surface and achieve the effect of demulsification by continuously gathering one phase of the oil or water. In addition, after the demulsification is completed, the formed simple oil–water mixture still needs to be further separated by the above two methods.

## 3. Two-Dimensional Materials in Oil–Water Separation

Two-dimensional materials are nanomaterials with sheet-like or layered structures. Their lateral size ranges from tens of nanometers to hundreds of micrometers, and their vertical thickness ranges from a single atom to several atoms. The preparation methods, characteristics, advantages, and drawbacks of different 2D materials are shown in Table 1.

### 3.1. MXene

As a two-dimensional transition metal-carbon/nitride, MXene has developed rapidly recently, and dozens of MXenes have been prepared. Due to its excellent electrical conductivity, MXene has been widely used in electronics [25] and electromagnetic shielding [26] fields. In addition, due to the unique layered loose structure and a large number of hydrophilic functional groups on the surface, MXene also has the possibility of application in the field of oil–water separation. However, there is still a lack of research in this area. Here, the preparation principle of MXene and the latest progress in oil–water separation are summarized.

**Figure 2:**
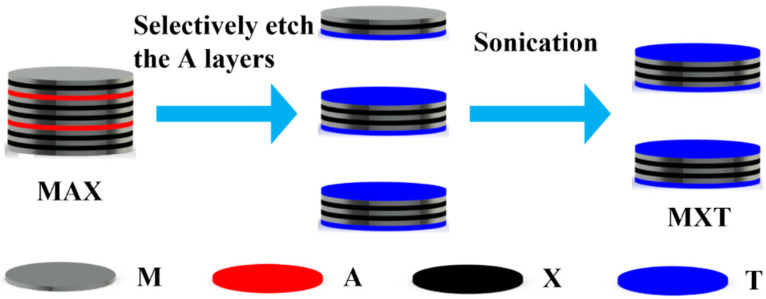
Schematic diagram of the preparation of MXene. M is an early transition metal, A is the main-group element, X is carbon/nitrogen, and T is the surface functional group.

The preparation mechanism of MXene has been pointed out since it was first prepared in 2011 [27]. Since the M-X bonds in the MAX phase are more stable and the chemical properties of A are more active, MXenes are prepared by selectively etching A in the MAX phase, as shown in Figure 2. Where M is an early transition metal, A is the main-group element, and X is carbon/nitrogen. For example, Ghidiu et al. etched titanium aluminum carbide (Ti_3_AlC_2_) in lithium fluoride (LiF) and hydrochloric acid (HCl) solutions, then, the MXene material was obtained by selective removal of Al and sonication [28]. In addition, the general formula of MXene can be represented by M_n+1_X_n_T_x_ [29], where T_x_ represents the surface functional group (O, OH, F).

The inherent hydrophilicity benefits of MXenes are very suitable for preparing underwater superoleophobic materials. However, affected by the preparation technology and process, untreated MXene generally exists in the form of dispersed nanosheets, so other materials need to be used as supporting substrates when preparing pure MXene oil–water separation membranes. Zhang et al. reported an MXene membrane with a high flux of 887 L m^−2^ h^−1^ bar^−1^ prepared by depositing Ti_3_C_2_T_x_ on a polyvinylidene fluoride (PVDF) membrane by a vacuum filtration process, which also exhibited excellent durability and could remove oil in corrosive solutions [30]. Similarly, Li et al. reported a membrane with a high separation efficiency of 99.94% for emulsion separation by using the more hydrophilic polyethersulfone (PES) as the support layer [31]. However, traditional flexible substrates are complex, expensive, and unsuitable for mass production. Starting from cost reduction and large-scale production, Saththasivam et al. prepared a robust and durable oil–water separation membrane based on commercial printing paper [32].

Many functional groups on the surface of MXene provide many active sites for modification treatment. With the deepening of research, how to modify MXene to obtain better performance has attracted the interest of scholars. Oil or water flux is one of the criteria to measure the performance of oil–water separation materials, then the void is the key factor for the flux [33]. Feng et al. prepared the reduced graphene oxide (RGO)/polydopamine (PDA)/titanium carbide (MXene) composites by the dopamine modification method [34]. The incorporation of MXene in the membrane increased the interlayer spacing, thereby increasing the flux of the membrane. Similarly, Long et al. obtained a surface hydroxyl-rich MXene membrane by sodium hydroxide (NaOH) modification, which showed an emulsion flux as high as 6385 L m^−2^ h^−1^ bar^−1^ [35]. Besides membranes, powder demulsifiers are also commonly used in oil–water separation. Du et al. used Ti_3_C_2_T_x_ as a carrier to prepare MXene powder with a demulsification effect on strongly acidic emulsions after combining with chitosan [36].

**Figure 3:**
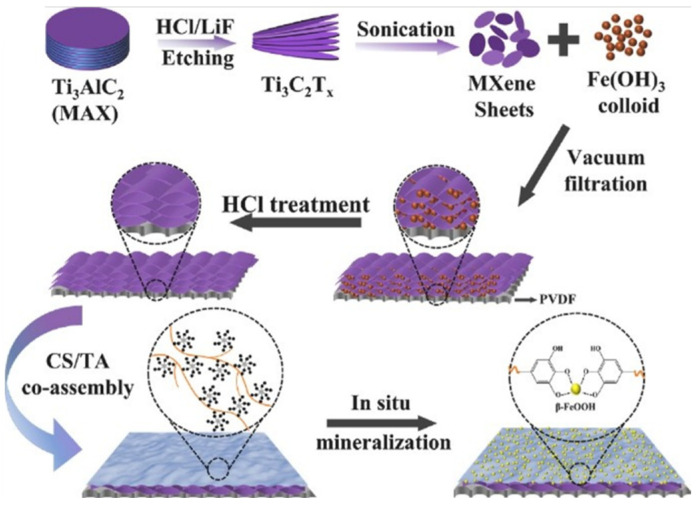
Schematic diagram of membrane preparation. Reproduced with permission from [42], Copyright 2021 Elsevier.

In addition to excellent performance, it is also crucial to have sufficient durability for practical applications. Here, durability mainly refers to the material’s pollution resistance, oxidation resistance, and chemical stability during the oil–water separation process. And previous studies have pointed out that organic pollution and surface oxidation are the main reasons for the change in the contact angle of MXene materials to water [37]. As the separation continues, pollutants such as proteins and small molecules in the oil–water mixture will gradually accumulate on the material’s surface. When a certain amount is reached, these pollutions can block the pores, resulting in reduced separation efficiency or failure. To solve this problem, He et al. added nanoparticles into MXene to prepare a separation membrane with high pollution resistance and chemical stability [38]. Compared with this passive anti-pollution method, the active anti-pollution strategy shows a better self-cleaning effect. Lin et al. revealed the mechanism of photocatalytic degradation of pollutants through density functional theory and finite element analysis [39]. On this basis, Feng et al. prepared a composite membrane with excellent photocatalytic degradation to various dyes through the synergy effect of MXene and titanium dioxide (TiO_2_) [40]. At the same time, it is necessary to improve the oxidation resistance of MXene, because the exposed transition metals in MXene are easily oxidized, resulting in the degradation or disappearance of the separation performance. Zeng et al. used nanotubes and PDA to modify MXene synergistically, and the reducibility of PDA was demonstrated to inhibit the oxidation of MXene [41]. In addition, oil–water separation materials with excellent comprehensive properties are the future direction of development. Hu et al. prepared separation membranes with high pollution resistance, corrosion resistance, reusability, and self-cleaning effect by self-assembling MXene on PVDF substrates, as shown in Figure 3 [42].

Although MXene is very promising for preparing underwater superoleophobic materials due to the existence of hydrophilic functional groups on the surface, there are still some problems to be solved. On the one hand, thanks to the unique layered structure, the voids between the layers enable MXene to exhibit excellent performance in separating oil–water emulsions. However, due to the easy oxidation of exposed transition metals, MXenes are challenging to be used in water/oxygen environments for a long time, which is also one of the main limitations of MXene materials. On the other hand, MXenes use a lot of strong corrosive and fluorine-containing chemical reagents in the preparation process. This poses a safety hazard to the operator and harms the environment, so how to prepare MXenes in a safer and greener way is also one of the issues to be considered.

### 3.2. 2D MOF

A metal–organic framework (MOF) is a new porous material usually formed by linking organic ligands with metal ions as nodes. Since the first synthesis of a MOF by Yaghi et al. [43], a large number of MOFs have been synthesized over the decades and have been widely used in catalysts [44], sensors [45], and water treatment [46]. With the advent of graphene, 2D MOFs have aroused extensive interest among scholars.

**Figure 4:**
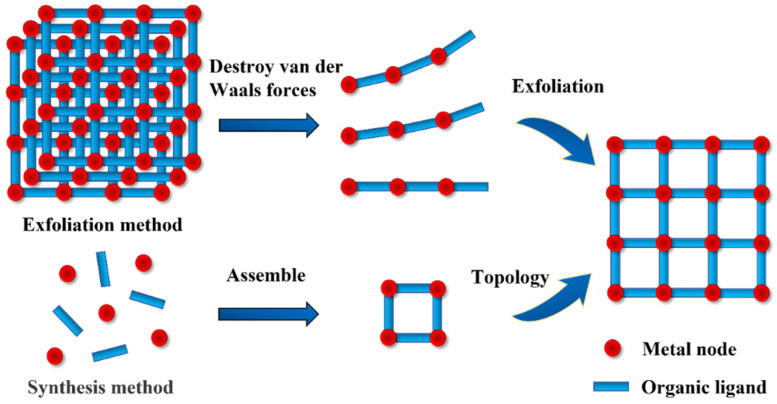
Top-down exfoliation and bottom-up synthesis.

The current methods for synthesizing 2D MOFs are mainly divided into the top-down exfoliation method and the bottom-up synthesis method [47], as shown in Figure 4. A 2D MOF is composed of a single layer or several layers of atoms or molecules, which are connected by chemical bonds within the layers and connected by intermolecular force between the layers. Since the interlayer intermolecular force is much weaker than the intralayer chemical bond, it can be broken by applying a simple force (such as ultrasound, etc.) to exfoliate the layered 2D MOF from the bulk MOF. Sang et al. prepared a 2D MOF by ultrasonically exfoliating bulk MAMS-1 crystals in a deep eutectic solvent with a maximum exfoliation rate of up to 70% [48]. Liu et al. reported a single-layer 2D Zn-MOF with a thickness of only 3.4 nm, which took the method of further weakening and destroying the interlayer force before exfoliation [49]. Unlike the exfoliation method of forcibly exfoliating 2D MOFs from existing 3D MOFs, the bottom-up synthesis method is prepared through direct reactions between metal nodes and organic ligands. The synthesis methods are divided into the solvothermal method [50], the catalyst-assisted method [51], and the assembly method [52], etc. Among them, the solvothermal method is the most widely used. Due to the large specific surface area of 2D materials, it is necessary to limit the growth in one direction during the synthesis of nanosheets [53]. Jia et al. prepared a 2D NiFe MOF by a solvothermal method [54]. They found that the solvent composition had a significant influence on the morphology and properties of a NiFe MOF, and different solvent mixtures have different inhibitory effects on organic ligands growing along the same direction. In addition to the two common preparation methods mentioned above, new methods are developed on this basis. Deng et al. reported a ligand replacement strategy to preparing a monolayer 2D MOF with excellent oil–water separation performance from a 3D MOF by replacing the original ligands with stronger ligands binding to metal nodes [55].

In MOFs, the structure and geometry formed by metal nodes and organic ligands play a crucial role in the material’s performance, and an excellent separation effect can be achieved for oil–water emulsions by constructing a reasonable structure and size of pores. Gai et al. reported a topologically structured 2D kgd-Zn MOF prepared by a reticular chemical strategy and solvothermal method [56]. The results showed that the as-prepared 2D kgd-Zn MOF has ultra-high porosity and chemical stability, offering excellent application prospects in oil–water separation. In addition, different from the active sites of 3D MOFs that mainly exist inside the pores, the active sites of 2D MOFs are usually exposed on the material surface, which lays the foundation for the application of 2D MOFs in oil–water separation. The 2D MOFs can be endowed with different functions by rationally deploying functional groups on the organic ligands, for example, the carboxyl and amino groups determine the hydrophilicity/hydrophobicity of the material, respectively. Gao et al. grew a 2D Ni-Fe MOF on the surface of halloysite nanosilica-alumina tubes (HNTs) by in situ hydrothermal synthesis [57]. Benefiting from the carboxyl groups of the 2D Ni-Fe MOF, the material exhibits super-hydrophilicity, with the separation efficiency and flux for oil–water emulsions reaching 98% and 970 Lm^−2^ h^−1^, respectively.

Compared with traditional porous materials, MOFs have the advantages of controllable pore size and structure. By selecting suitable metal nodes and organic ligands, small and uniform pore sizes can be obtained after a rational design of its structure. Two-dimensional MOFs are oil–water separation materials with good application prospects, but problems are still to be solved. First, during the oil–water separation process, the organic ligands will be gradually replaced by water molecules, eventually leading to the overall structure’s collapse. Therefore, how to improve the stability of 2D MOFs requires further research. Second, although the existing methods have improved the yield, they are still insufficient to meet the actual production needs. The large-scale, low-cost, and rapid preparation of 2D MOFs is one of the challenges in the future.

### 3.3. 2D COF

A covalent organic framework (COF) is also a porous material with topological structures, first synthesized by Yaghi’s group in 2005 [58]. As the synthesis inspiration of a COF came from a MOF, it also has the characteristics of a high specific surface area and strong structure controllability. The difference is that the framework of the COF is formed by covalent bonds between light elements (C, N, O, H, B, Si, etc.). Currently, COFs are mainly divided into three types: boron-containing [59], imine [60], and triazine [61]. And they have applications in seawater desalination [62], oil–water separation [63], microcapsules [64], and other fields. Like a 2D MOF, the preparation methods of a 2D COF are mainly divided into the top-down exfoliation method [65] and the bottom-up synthesis method [66], so only the topology of a 2D COF is introduced here.

**Figure 5:**
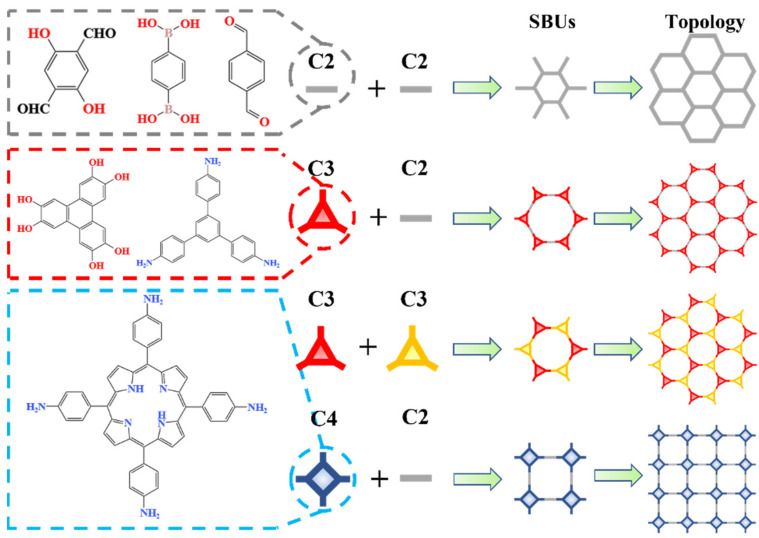
Topological methods for 2D COF.

First, the precursors of a 2D COF can be divided into three types: C2, C3, and C4. Among them, C2 represents the precursor with reactive sites at the symmetrical ends of the chemical structure, while C3 and C4 represent the precursors with reactive sites on the three vertices of the triangle or the four vertices of the square. As shown in Figure 5, they form secondary building units (SBUs) through the reaction between the precursors, and then 2D COFs with a network structure can be obtained from the topology of SBUs. Therefore, the common topological methods of a 2D COF can be divided into four types: C2 + C2, C2 + C3, C3 + C3, and C2 + C4. Since other topological methods (C2 + T4, etc.) belong to the construction methods of 3D COFs or do not meet the mesh strategy, they will not be introduced here.

To expand the application of COFs in different fields, it is essential to realize the functionalization of materials by introducing other functional groups into COFs [67]. For example, the adsorption capacity of mercury in an aqueous solution can be significantly improved by introducing sulfur elements in the framework of COFs [68]. Correspondingly, introducing hydrophilic/hydrophobic functional groups into the COF framework can realize the application in oil–water separation. Since hydrophobic materials generally have better pollution resistance, it is more appropriate to introduce hydrophobic functional groups. Chen et al. prepared an adsorbent with an emulsion separation efficiency higher than 99.5% by introducing fluorine into PDA [69]. Introducing low surface energy fluorine groups brings superhydrophobicity and stability to the 2D COF powder, which has a good application prospect in oil–water separation. To further enhance the application of a 2D COF in oil–water separation, Liu et al. introduced fluorine groups into both precursors and prepared 2D COF coatings by direct growth on a stainless steel mesh [70]. The results show that the permeation flux of the material is as high as 2.84 × 105 L m^−2^ h^−1^, which is one of the highest values reported so far.

A 2D COF has the characteristics that the structure, porosity, and pore size can be designed in advance and has certain application prospects in oil–water separation, but it is still in its infancy. Because of the long-term contact with water, the water stability of a COF is also one of the problems that must be solved, just like a MOF. For this reason, the COFs of imine and triazine perform better. Secondly, whether it is a COF or a MOF, the preparation process often takes several days. How to control the cost and improve production efficiency is the premise of a wide range of applications.

### 3.4. LDH

**Figure 6:**
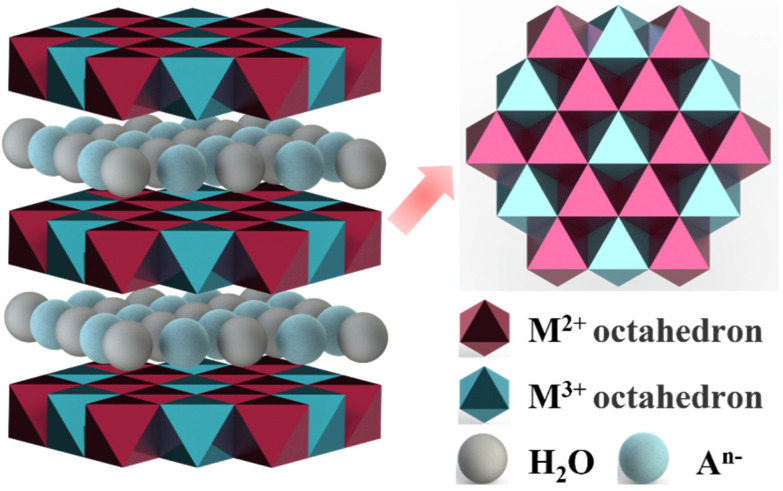
The typical structure of LDH.

Layered double hydroxide (LDH) is a two-dimensional anionic layered material, also known as a hydrotalcite-like compound. LDH is composed of the main layer and interlayer anions. And the main layer is generally composed of two metal cations, each metal cation is coordinated with six hydroxyl groups to form an octahedron, and each octahedron shares edges, as shown in Figure 6. Therefore, the general chemical formula of LDH can be written as $[M_{1-x}^{2+}M_{x}^{3+}{\left(OH\right)}_{2}]{\left[A^{n-}\right]}_{\frac{x}{n}}\cdot mH_{{}_{2}}O$, where *M*^2+^ represents a divalent metal cation (Mg^2+^, Ni^2+^, Cu^2+^, Zn^2+^, Co^2+^, etc.), *M*^3+^ represents a trivalent metal cation (Al^3+^, Fe^3+^, Ga^3+^, Mn^3+^, Gr^3+^, etc.), *A*^*n*−^ represents an interlayer anion(Cl^−^, NO^3−^, CO_3_^2−^, SO_4_^2−^, etc.), and the value of *x* is the molar ratio of *M*^2+^ to *M*^3+^ (0.2–0.4) [71]. Due to the properties of interlayer anion exchangeability, thermal stability, and unique memory effect, LDH has applications in antibacterial [72], catalyst [73], gas separation [74], and other fields.

Currently, the main preparation methods of LDH include coprecipitation, hydrothermal synthesis, and anion exchange. The coprecipitation method is the earliest and most common method for preparing LDH, which has the advantages of high crystallinity and simple operation. Adding an alkaline precipitant (NaOH, etc.) to the metal salt solution, LDH can be obtained after the precipitate is precipitated, washed, and dried. Li et al. obtained MgAl-LDH after adding aqueous ammonia to the mixed solution containing MgCl_2_ and AlCl_3_, which can be used for oil–water separation [75]. However, the LDH produced by the coprecipitation method is formed sequentially, leading to different sizes. The proposal of the hydrothermal method makes up for this defect. Different from the coprecipitation method, the hydrothermal method rapidly transfers the mixed solution into an autoclave, and synthesizes small and uniform LDH under high temperature and high pressure. Feng et al. added a mixed solution containing Fe(NO_3_)_3_ and Ni(NO_3_)_2_ into an autoclave and heated it at 120 °C for ten hours to obtain 2D NiFe-LDH [76]. In addition, the anion exchange method obtains a new LDH by replacing the original interlayer anions. The common sequence of anion replacement is as follows: CO_3_^2−^ > SO_4_^2−^ > HPO_4_^2−^ > OH^−^ > F^−^ > Cl^−^ > Br^−^ > NO_3_^−^ > I^−^. Wu et al. successfully achieved the intercalation of vanadate in the as-prepared MgAl-LDH through the anion exchange method [77]. Because of the harsh preparation conditions, this method is rarely used.

**Figure 7:**
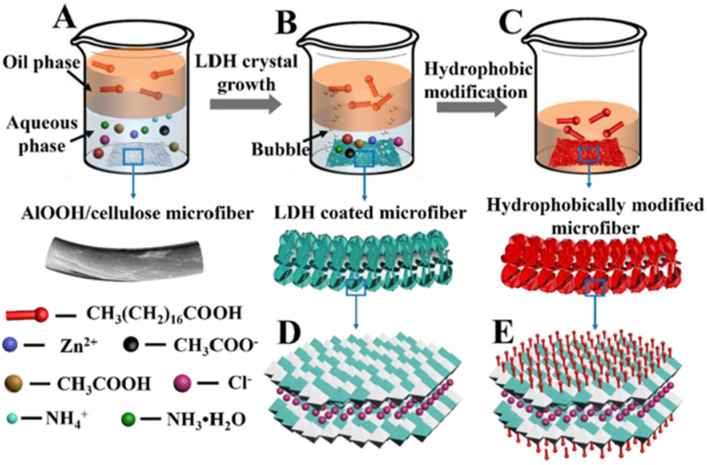
(**A–E**) The combination of the hydrothermal method and hydrophobic modification. Reproduced with permission from [81], Copyright 2021 Elsevier.

Different functions can be obtained by changing the type and proportion of metal cations in the main laminates of LDH. Since LDH does not have a pore or gap structure, it is not an optimal oil–water separation material. Still, it is feasible to fabricate microscopic roughness through LDH to achieve different wettability. Cui et al. grew NiCo-LDH with a grass-like structure on the PVDF membrane by the hydrothermal method, and the obtained composite membrane exhibited superhydrophilicity/underwater superoleophobicity for separating both surfactant-free and surfactant-stabilized oil–water emulsions by a capillary effect under the driving of gravity [78]. However, the pollution resistance of the membrane should also be considered. In this regard, hydrophobic materials have the advantage of a self-cleaning effect. Aladpoosh et al. used a 2D MgAl-LDH to construct micro-roughness on cotton fabric fibers and subsequently achieved superhydrophobic modification of the cotton fabric by dipping it in a stearic acid solution [79]. Similarly, Wang et al. used PDMS to coat cotton fabric with AlNi-LDH to obtain a superhydrophobic surface, and the modified cotton fabric showed excellent pollution resistance and 96% oil–water separation efficiency [80]. To simplify the preparation process, Yue et al. combined the hydrothermal method and the modification process to prepare superhydrophobic filter paper, as shown in Figure 7 [81]. In the aqueous phase, the rough surface of cellulose fibers is formed by utilizing controlled crystal growth, while the superhydrophobic surface is obtained by using the self-assembly of stearic acid in the oil phase. The as-prepared superhydrophobic LDH/cellulose membrane shows outstanding separation performance with extremely selective absorption. This method is simple, versatile, reproducible, can be extended to other oil–water separation materials, and has a promising application prospect.

With the in-depth research of LDH in oil–water separation, scholars have begun to try to synthesize ternary metal cation LDH [82]. Sun et al. grew ternary ZnNiCo-LDH on stainless steel mesh and then prepared stainless steel mesh with photocatalytic degradation ability after combining it with NiMoO_4_ nanosheets [83]. The modified stainless steel mesh has an underwater oil contact angle of 164.9° and maintains 99.9% separation efficiency after 60 separation cycles. Similarly, Zhang et al. prepared a MgNiAl-LDH adsorbent with magnetic response properties for oil–water separation by the coprecipitation method [84].

In addition to the metal cations in the main layer, different combinations of interlayer anions can also realize the multi-functionalization of materials. Still, there is no relevant report on LDH in oil–water separation materials. In addition, the exchangeability of the anion layer of LDH will also weaken the acid and alkali resistance of the material, and how to maintain the stability of LDH deserves further research.

### 3.5. Graphene

**Figure 8:**
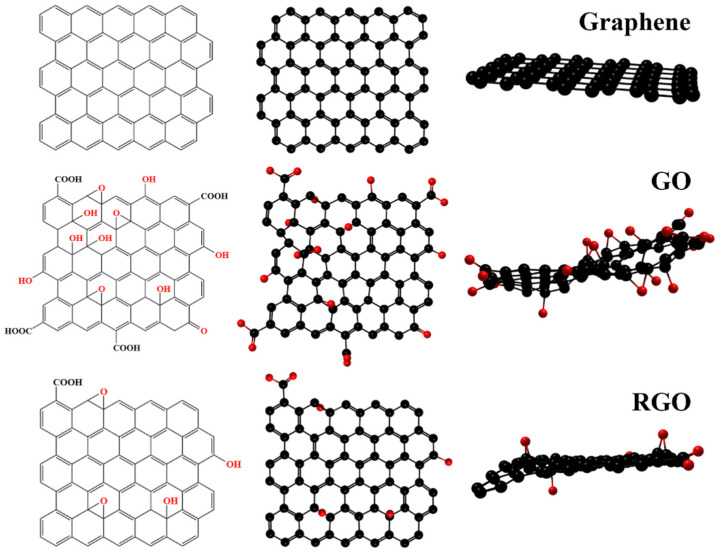
Schematic diagram of the structure of graphene and its derivatives.

Since its inception, graphene, the first two-dimensional material, has attracted much attention. There are many studies and reviews on graphene and its derivatives [85,86,87]. Figure 8 shows the structure of graphene and its derivatives. With the efforts of scholars, graphene has shown excellent potential in various fields, including oil–water separation [88]. However, the surface of graphene does not contain functional groups, which is disadvantageous for the functionalization of graphene. In contrast, graphene oxide (GO) with many oxygen-containing functional groups on its surface has a better application prospect in oil–water separation [89,90]. First, these oxygen-containing functional groups are almost all hydrophilic, which can efficiently and selectively separate water under the action of hydrogen bonds and possess a high water flux [91]. For example, Cai et al. prepared a GO-based membrane capable of separating oil-in-water emulsions, and the membrane water flux was as high as 4600 L m^2^ h^−1^ bar^−1^ [92]. Second, a large number of functional groups provide sites for subsequent hydrophobic modification or functionalization, which can further enhance the performance of materials, including efficiency and durability [93,94]. Zhou et al. adhered the modified GO to a sponge’s surface and prepared a sponge for oil–water separation that can separate oil 53,000 times its own mass with an efficiency higher than 99.5% [95].

The existence of functional groups makes GO hydrophilic easy to disperse but also affects its stability. Although graphene has excellent stability, it is not suitable for large-scale application in the field of oil–water separation due to the difficulties in preparation and functionalization currently. Therefore, reduced graphene oxide (RGO) as an intermediate product is the best choice [96]. First, RGO is obtained by GO under the action of a reducing agent, so the oxygen-containing functional groups on the surface are significantly reduced, which improves the stability of RGO [97]. Moreover, the reduction of functional groups enhances the hydrophobicity of RGO, making it easier to construct materials for oil–water separation with strong pollution-resistance properties. Zhan et al. reported a MOF@RGO superhydrophobic hybrid material with a high water contact angle of 169.3 ± 0.6° and high stability [98]. Second, the residual functional groups on the surface of RGO reserve sites for subsequent functionalization and modification, showing stronger scalability than graphene [99]. In addition, the residual functional groups are also favorable for the bonding between RGO and the substrate material. Song et al. reduced GO to RGO by ultrasonic-microwave synergy and fixed it firmly on the sponge, and the obtained material showed good reusability and rapid acquisition [100].

Graphene and its derivatives with excellent oil–water separation performance are potential materials. Considering the cost and performance comprehensively, RGO among them seems to be the most suitable for preparing oil–water separation materials, but there are contents worthy of discussion. Among them, it is significant to control the number of functional groups on the surface of RGO to achieve functionalization and strong stability simultaneously.

### 3.6. Two-Dimensional TMD

**Figure 9:**
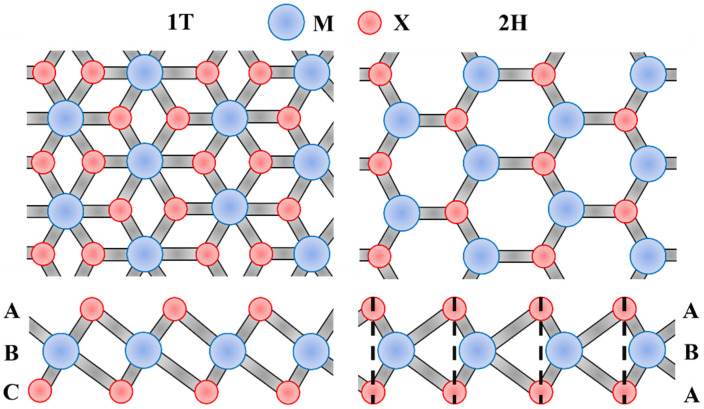
Top and side views of the ABA and ABC stacking modes of the atoms of MX_2_.

Transition metal dichalcogenide (TMD) is a graphene analog with a “sandwich” structure, which has been widely studied and applied in the fields of optics [101] and electronics [102]. The general formula for TMD can be written as MX_2_, where M represents a transition metal atom, and X represents a chalcogen. The stacking modes of the atoms of MX_2_ are ABA and ABC, corresponding to the triangular prism coordination (2H) and the octahedral coordination (1T), respectively, as shown in Figure 9. In the ABA stacking mode, the bottom atoms are facing the top atoms, while in the ABC stacking mode, they are staggered.

Molybdenum disulfide (MoS_2_) is the most typical and studied 2D TMD material. For example, Othman et al. reported an RGO-functionalized MoS_2_ membrane, which can remove oil from wastewater [103]. However, the application of 2D MoS_2_ usually requires attachment to a substrate, especially in oil–water separation. Common porous substrates for separation materials include fabrics [104], metal meshes [105], sponges [106], and so on. Yu et al. dip-coated MoS_2_ on a sponge and then prepared an oil–water separation sponge by diazotization reaction [107]. The obtained sponge is superhydrophobic and exhibits excellent stability in harsh environments. Besides MoS_2_, other TMD materials have also been applied in oil–water separation [108]. Zhai et al. prepared a tungsten disulfide (WS_2_)-attached sponge by a one-step dipping method. The resulting sponge had a water contact angle of 158.8° and a separation efficiency higher than 99.85% [109]. In addition, blending different types of TMDs can sometimes provide a gain. Krasian et al. prepared a fiber mat blended with MoS_2_ and WS_2_, and the blending of nanosheets increased the surface roughness of the material, which significantly improved the hydrophobicity of the material [110]. At the same time, the attachment of the two nanosheets provided more oil adsorption sites for the fiber mat, which increased the material’s oil adsorption capacity by 190%.

As a 2D material that is expected to be a substitute for graphene, TMD possesses semiconducting or superconducting properties [111], which is a very potential material. However, the research on 2D TMD is still in its infancy, and there are few reports in the field of oil–water separation. At present, the research of TMD is mostly focused on MoS2 and WS2, and the types of oil–water separation materials should be enriched.

## 4. The 2D to 3D Transformation Method

**Figure 10:**
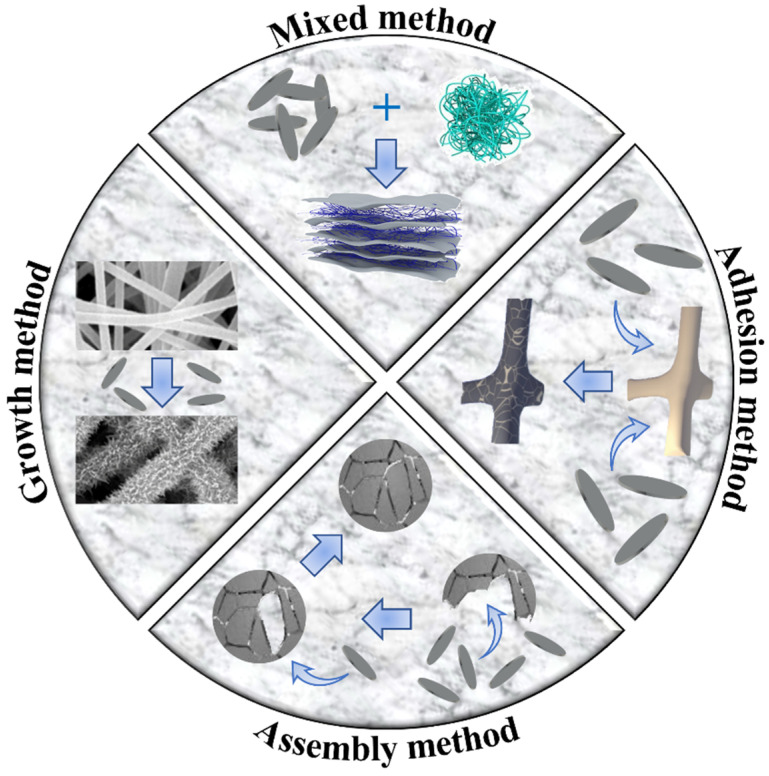
Four methods from 2D to 3D.

Currently, 2D materials cannot be prepared at wafer-level size, which still exist in the form of powder on a macroscopic scale. These powders can have a demulsification effect on oil–water emulsions through processing. However, they still cannot continuously separate oil–water mixtures and emulsions, which greatly limits the application of 2D materials in oil–water separation. To overcome this limitation, it is usually necessary to transform 2D materials into materials with 3D structures on a macroscopic scale, such as membranes, sponges, foams, and other materials. This section introduces four methods for transforming 2D materials into 3D materials, including the mixed, adhesion, growth, and assembly methods, as shown in Figure 10.

### 4.1. Mixed Method

The mixed method is the most straightforward 2D-to-3D method, and materials with 3D structures can be obtained by simply doping 2D materials into other materials. Although the principle and preparation of the mixed method are straightforward, the compatibility of materials needs to be carefully considered when selecting materials. For example, Liu et al. prepared a composite aerogel with oil–water separation ability after freeze-drying and thermal imidization of a solution containing MXene and polyimide (PI) fibers [112]. Similarly, Wang et al. added the polyacrylic acid powder to the MXene suspension and obtained PI/MXene aerogel after the same treatment [113]. In addition, the unique advantages of 2D materials come from their large specific surface area and atomic-scale thickness, but the mixed method would largely waste these features. The mixed 2D materials are either aggregated together or embedded in other materials, making it difficult to exhibit their properties. To take full advantage of the characteristics of single-layer 2D materials, Lu et al. fabricated carbon aerogels using one-dimensional nano-fibrillated cellulose as the interlayer of GO [114]. Since the fibrillated cellulose acts as a support between the single-layer GO nanosheets, the GO nanosheets are not stacked together and the layered structures are largely preserved. In addition, the mixed method can also be used for the preparation of the membrane. Ajibade et al. prepared a mixed solution of MXene, functionalized multi-walled carbon nanotubes, and polyacrylonitrile into a membrane by the phase inversion method, and the obtained membrane has good stability [115].

### 4.2. Adhesion Method

The adhesion method is one of the ordinary and convenient methods in the material preparation process, which can also realize the transformation of 2D materials to 3D materials [116]. The adhesion method can realize the adhesion of nanosheets on the surface of materials by various methods such as dip coating, spray coating, chemical vapor deposition, and vacuum filtration [117,118]. And this adhesion is formed under the action of hydrogen bonds, chemical bonds, or intermediate substances. Hu et al. reported a bacterial cellulose/PDA/RGO ultrafiltration membrane that can be used for long-term oil–water separation, in which PDA acts as an intermediate to attach RGO onto the surface of the membrane [119]. However, the adhesion method has a problem that cannot be ignored. That is, the 2D nanosheets attached to the surface of the material are easily worn or peeled off. Wang et al. first attached MXene nanosheets to wood sponges by freeze-drying and then immersed them in PDMS solution to prepare sponges with excellent mechanical properties [120]. Benefiting from the coating of PDMS, the sponge was hydrophobic, and the nanosheets were protected. In addition, the adhesion method has a low threshold and a wide range of applications, which is suitable for different 2D materials [121].

### 4.3. Growth Method

Unlike the adhesion method, which needs to be attached to the substrate from top to bottom, the growth method can directly grow nanosheets on the substrate from the bottom to the top. The growth method has been widely used in preparing 2D nanosheets due to its simple process and easy operation [122]. For 2D materials, the growth method mainly refers to hydrothermal, through which highly ordered structures can be in situ grown on the substrate. For example, Shami et al. have grown vertically aligned, robust MgAl-LDH nanosheets with the 3D rough structure on electrospun polyacrylonitrile (PAN) membranes in a large area [123]. And because these nanosheets are grown on substrates, they usually exhibit higher mechanical and chemical stability than the adhesion method [124]. Li et al. grew caterpillar-like nanosheets on PAN membranes, and the prepared membranes could be washed with a small amount of solvent, showing excellent durability [125].

With the help of the growth method, the 2D material builds a rough microscopic structure for the substrate, and the rough microscopic structure can make the material surface more hydrophilic or hydrophobic. Since 2D materials are hydrophilic inherently, these materials tend to exhibit superhydrophilicity. Superhydrophilicity is beneficial for materials to obtain large water flux. However, with the progress of oil–water separation, pollutants (metal ions, etc.) in water will be gradually adsorbed by active sites on 2D materials, eventually blocking pores. However, superhydrophobic materials have outstanding performance in pollution resistance, so it is necessary to modify 2D materials hydrophobically. Zhang et al. grew NiAl-LDH on stainless steel meshes, which were subsequently hydrophobically modified with low surface energy substances [126]. The mesh modified by low surface energy substances has self-cleaning properties. The surface wettability changes from hydrophilic to superhydrophobic, and the water contact and rolling angles are 156° and 5°, respectively.

### 4.4. Assembly Method

The assembly method is a method that structural units (atoms, molecules, nanomaterials, etc.) change from disorder to order under intermolecular force or additional external force to form a stable and geometric structure. For example, He et al. reported a hydrogen bond-mediated self-assembled graphene aerogel [127]. Common assembly methods include but are not limited to the liquid–liquid interface assembly method [128], the freeze-drying assembly method [129], the electrostatic self-assembly method [130], etc. Compared with the growth method, the assembly method has the advantages of a controllable process and structure. Cai et al. prepared MXene aerogels by controlling the growth of ice during freeze-drying, which had the characteristics of a controllable microstructure and multifunctionality [131]. Electrostatic self-assembly is a technique that uses electrostatic interactions between opposite electric charges to assemble substances. For example, Zhao et al. utilized the electrostatic interaction between negatively charged MXene and positively charged polystyrene (PS) chains to selectively graft PS onto one side of MXene to form Janus nanosheets [132]. Similarly, Nikzad et al. formed a GO-LDH hybrid membrane on a stainless steel mesh by self-assembly through the electrostatic interaction between negatively charged GO and positively charged LDH [133]. The resulting mesh showed an oil–water separation efficiency greater than 99.5% with a gravity-driven water flux of 160,222 ± 102 L m^2^ h^−1^.

Table 2 shows the above four methods’ characteristics, advantages, and drawbacks.

## 5. Conclusions

Since its first preparation, 2D materials represented by graphene have received extensive attention. Benefiting from atomic-scale thickness, large specific surface area, and unique structure, 2D materials exhibit excellent optical, electrical, mechanical, and thermal properties. With the deepening of research, the types and application fields of 2D materials have been greatly expanded in the past decade. Although 2D material is a highly potential and revolutionary material, its research is still in the early stage, and it has not been able to achieve large-scale and practical applications. Especially in oil–water separation, the research and application of 2D materials are still in their infancy, and many problems are still to be solved.

First, for 2D materials to be practically applied in oil–water separation, stability is a problem that must be solved. Since the surface of 2D materials usually contains many oxygen-containing functional groups, they are prone to hydrolysis in a humid environment. However, the separation of oil and water depends on these functional groups largely, and how to precisely control the number and types of surface functional groups needs further research. Second, in the process of oil–water separation, one of the oil or water phase will be accumulated on one side of the material, and contaminants will also be accompanied by the accumulation of liquid. Since the active sites of 2D materials are exposed, these contaminants are easily adsorbed and blocked, eventually resulting in material failure. In addition, liquid accumulation will also cause the pressure on the material surface to rise. When the pressure exceeds a certain value, the accumulated liquid will be forced to pass through the material and pollute the separated liquid. Therefore, the issues of pollution resistance and liquid drainage should be considered when designing 2D oil–water separation materials. Third, the research results of 2D oil–water separation materials at present are almost based on the laboratory, it is feasible to separate oil–water mixtures with small areas and small flow rates. However, it is worth considering whether the large area and large flow still have excellent results in actual industrial emissions. Fourth, a large pore size usually means a large flux in oil–water separation, but it is accompanied by lower separation efficiency, and a small pore size is the opposite. The material can have both efficiency and flux by designing a reasonable pore size and porosity. However, it is still difficult to accurately control the structure of 2D materials and the proportion of a specified phase. Fifth, 2D materials in oil–water separation are mostly in a powder state macroscopically. Even if a small part can show the morphology of the membrane macroscopically, it is mainly achieved by simple stacking. This will cause the unique properties of 2D materials not to be fully utilized, such as atomic thickness and large specific surface area, which will be significantly weakened after stacking. Therefore, there is still a lack of a method that can manufacture and grow wafer-scale 2D materials on a large scale. In this regard, the assembly method may be a shortcut, but precisely controlling the assembly process of 2D materials is a huge challenge. Finally, at present, the use of toxic and harmful chemicals, especially fluorine-containing reagents, will still be a problem that cannot be ignored in the field of oil–water separation. Therefore, there is a long way to go to develop easily available and environment-friendly oil–water separation materials.

## Figures and Tables

**Table 1:** The preparation methods and characteristics of 2D materials and their advantages and drawbacks in oil–water separation.

Classification	Preparation Methods	Features	Advantages	Drawbacks	References
MXene	Selectively etching A in the MAX phase	Unique layered loose structure	Many functional groups Many voids	Easily oxidized	[25,26,27,28,29,30,31,32,33,34,35,36,37,38,39,40,41,42]
2D MOF	Exfoliation method Synthesis method	Porous structure Exposed active points Topological structures	Controllable pore size and structure Easy to modify	Poor water stability Long preparation time	[43,44,45,46,47,48,49,50,51,52,53,54,55,56,57]
2D COF	Exfoliation method Synthesis method	Porous structure Topological structures	Strong structure controllability	Poor water stability Long preparation time	[58,59,60,61,62,63,64,65,66,67,68,69,70]
LDH	Coprecipitation method Hydrothermal synthesis method Anion exchange method	Interlayer anion exchangeability Thermal stability Memory effect	Multifunctional property	No pore or gap structures Poor stability	[71,72,73,74,75,76,77,78,79,80,81,82,83,84]
Graphene and its derivatives	Exfoliation method Synthesis method Redox method	Many oxygen-containing functional groups Excellent thermal Conductivity Excellent electric conductivity Excellent light transmittance	Good hydrophilicity Easy to disperse	Poor stability	[85,86,87,88,89,90,91,92,93,94,95,96,97,98,99,100]
2D TMD	Exfoliation method Synthesis method	”Sandwich” structure Superconducting properties	Good hydrophilicity	Poor stability	[101,102,103,104,105,106,107,108,109,110,111]

**Table 2:** The technologies used by the four methods of 2D to 3D and their advantages and drawbacks.

2D to 3D Methods	Technology and Method	Advantages	Drawbacks	References
Mixed method	Mix	Simple principle Simple preparation	Compatibility problem Weaken the characteristics and advantages of 2D materials	[112,113,114,115]
Adhesion method	Dip coating Spray coating Chemical vapor deposition Vacuum filtration	Convenient Low Threshold Wide Application range	Poor stability	[116,117,118,119,120,121]
Growth method	Hydrothermal	Simple process Easy operation Good stability	Time consuming	[122,123,124,125,126]
Assembly method	Liquid–liquid interface assembly Freeze-drying assembly Electrostatic self-assembly	Controllable synthesis process Controllable structure	Complex process	[127,128,129,130,131,132,133]

## Data Availability

Data will be made available on request.
